# Free Layer Thickness Dependence of the Stability in Co_2_(Mn_0.6_Fe_0.4_)Ge Heusler Based CPP-GMR Read Sensor for Areal Density of 1 Tb/in^2^

**DOI:** 10.3390/mi12091010

**Published:** 2021-08-25

**Authors:** Pirat Khunkitti, Apirat Siritaratiwat, Kotchakorn Pituso

**Affiliations:** 1 KKU-Seagate Cooperation Research Laboratory, Department of Electrical Engineering, Faculty of Engineering, Khon Kaen University, Khon Kaen 40002, Thailand; apirat@kku.ac.th; 2 Seagate Technology (Thailand) Ltd., Muang 10270, Thailand; k.pituso@gmail.com

**Keywords:** magnetic recording, magnetic read heads, current perpendicular-to-the-plane giant magnetoresistance, Heusler alloys

## Abstract

Current-perpendicular-to-the-plane giant magnetoresistance (CPP-GMR) read sensors based on Heusler alloys are promising candidates for ultrahigh areal densities of magnetic data storage technology. In particular, the thickness of reader structures is one of the key factors for the development of practical CPP-GMR sensors. In this research, we studied the dependence of the free layer thickness on the stability of the Co_2_(Mn_0.6_Fe_0.4_)Ge Heusler-based CPP-GMR read head for an areal density of 1 Tb/in^2^, aiming to determine the appropriate layer thickness. The evaluations were done through simulations based on micromagnetic modelling. The reader stability indicators, including the magnetoresistance (MR) ratio, readback signal, dibit response asymmetry parameter, and power spectral density profile, were characterized and discussed. Our analysis demonstrates that the reader with a free layer thickness of 3 nm indicates the best stability performance for this particular head. A reasonably large MR ratio of 26% was obtained by the reader having this suitable layer thickness. The findings can be utilized to improve the design of the CPP-GMR reader for use in ultrahigh magnetic recording densities.

## 1. Introduction

As is widely claimed by several studies, current-perpendicular-to-the-plane giant magnetoresistance (CPP-GMR) devices have been promising candidates as the magnetic read sensors for ultrahigh areal densities (ADs) of magnetic data storage technology [1,2,3,4,5,6]. In the past few decades, the outstanding features of the CPP-GMR reader, i.e., large magnetoresistive (MR) outputs, extremely low resistance area (RA) product, capability of transferring large amounts of data at high speeds, and low thermal fluctuation, have been extensively proved [2,7,8,9]. The very low RA product of the CPP-GMR devices is a key factor in achieving significantly higher ADs than the tunnel magnetoresistance (TMR) junctions used in the current situation [10,11,12,13]. The CPP-GMR sensors based on ferromagnetic Heusler alloys are the most capable integrations that can provide very high performance of CPP-GMR sensors nowadays [14,15,16,17,18,19]. Therefore, several studies have attempted to improve the MR output of the CPP-GMR sensors using various Heusler alloy compositions; however, recent studies indicate that using the Co_2_(Mn_0.6_Fe_0.4_)Ge (CMFG) Heusler alloy as the sensing layer electrodes could provide the highest MR output [20,21,22,23].

At ultrahigh ADs in which the media bits must be rapidly downsized, the physical size of the reader needs to be reduced to prevent intertrack interference while maintaining adequate resolution [1,24,25,26]. The reader shield-to-shield spacing (SSS) is one of the structural parameters directly related to the physical dimension of the head. It also has a major impact on the down-track resolution. Therefore, reducing the SSS is a crucial point for increasing the AD. At an AD of 1 Tb/in^2^, the SSS was expected to be less than 25 nm [26]. In particular, the thickness of reader layers is a relative sizing parameter of the SSS. A few nanometers of layers embedded in reader structures are typically desired for the development of practical CPP-GMR read sensors, especially at higher ADs. It is well-known that the thickness of the reader layers also has an influential impact on head performance, particularly the stability of head response [14,27,28]. Thus, the suitable thickness of the head layers should be precisely designed for each one. 

In this work, we studied the dependence of the free layer thickness of the CPP-GMR reader on the head’s stability performance. The CPP-GMR head based on the CMFG Heusler alloy was focused, assuming that the head was targeted for AD of 1 Tb/in^2^. Micromagnetic simulations were based on the finite element method using the M3 code [29]. The rest of this paper is arranged as follows: CPP-GMR modelling is shown in Section 2. Section 3 describes the analysis of read head response. The simulation results, including the related discussions, are given in Section 4. Finally, the results are concluded in Section 5. 

## 2. CPP-GMR Modelling

**Figure 1:**
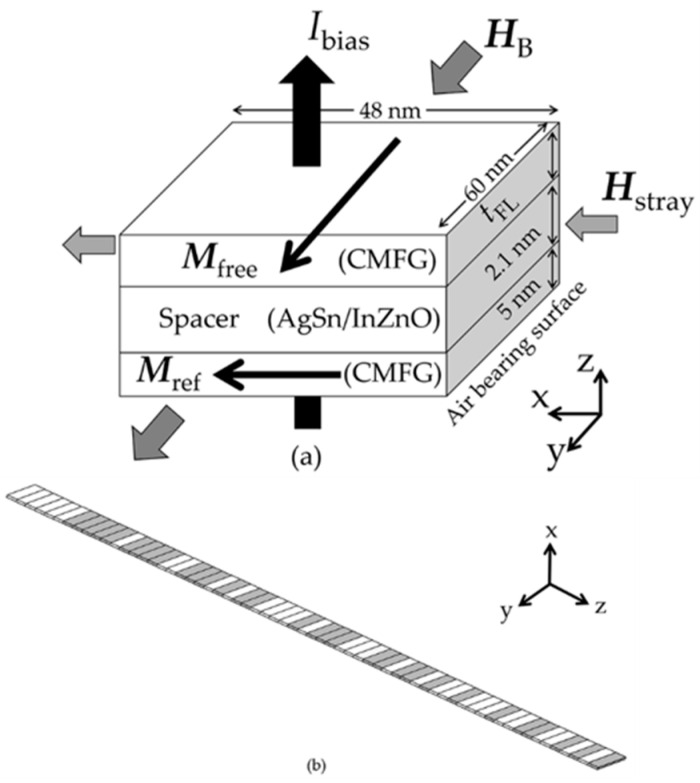
(**a**) CPP-GMR model and (**b**) 63 randomly generated media bits.

As shown in Figure 1a, the sensing layers of the CPP-GMR read head targeted for AD of 1 Tb/in^2^ are modelled, assuming that the head is sensing the magnetic stray field, ***H***_stray_, of the medium. The reader width and stripe height of the head were set at 60 and 48 nm, respectively, since this dimension was claimed as the appropriate value for the CPP-GMR reader at an AD of 1 Tb/in^2^ [30]. The combination of the CMFG electrodes and AgSn/InZnO spacer was performed due to their suitability for practical CPP-GMR devices [15]. The thickness of the bottom reference layer was 5 nm, while that of the spacer was 2.1 nm. The free layer thickness, *t*_FL_, was the main variable in this study, it was varied from 1 to 10 nm. The magnetization of the free layer, ***M***_free_, was along its easy axis (+*y*-axis), while the magnetization of the reference layer, ***M***_ref_, was fixed along the +*x*-axis, assuming that it is due to the exchange bias effect of the anti-ferromagnetic layer. The ***H***_stray_ produced by the medium was applied to the head on the air bearing surface (*x*-axis) to mimic the reading situation. The hard bias field, ***H***_B_, was uniformly supplied to the reader to provide a ±30° tilted angle of the free layer magnetization while receiving the ***H***_stray_. The magnetic media was assumed to be a perpendicular medium having a 10 nm hard layer, while a bit aspect ratio of 4 was set. The medium was based on FePt since it has been widely claimed as a promising material for overcoming the thermal stability limitation at high recording capacities [31,32]. The cross-track magnetic bits are shown in Figure 1b. Their sequence was generated by the 63 pseudorandom bit sequence (PRBS) using the *x*^6^ + *x*^5^ + 1 generator polynomial [33]. The gray- and white-filled bits indicate the direction of ***H***_stray_ along the +*x* and −*x* axis, respectively. It is noted that there are no writing errors or intertrack interference included in the simulations, therefore there is no transition noise. The head was assumed to be operated at 1 GHz for practical reasons.

The magnetic properties of CMFG Heusler alloy are adopted from reference [15], as follows: saturation magnetization of 10 × 10^5^ A/m, anisotropy constant of 8 × 10^3^ J/m^3^, spin polarization factor of 0.76, Gilbert damping parameter of 0.01, and exchange stiffness constant of 2.25 × 10^−11^ J/m. The RA product of the sensing layers was 0.11 Ωµm^2^. The head was biased with the bias current density of 1.96 × 10^6^ A/cm^2^, where its magnitude was purposely limited in order to minimize the influence of spin torque induced instabilities from this current. A positive sign of bias current is when it flows from the reference to the free layers. The device was assumed to be operated at room temperature. The time-varying magnetization was described using the Landau–Lifshitz–Gilbert–Slonczewski (LLGS) formula, as expressed in references [34,35]. A computational cell size of 2.5 × 2.5 × 2.5 nm^3^ and a time step of 0.1 ps were set in the simulations.

## 3. Analysis of Read Head Response 

It is well known that a few nanometers of thickness layers typically have an influential impact on the read head performance, especially at higher ADs. Therefore, the layer thickness of Heusler alloy films embedded in the CPP-GMR structure needs to be optimally designed to achieve the desired physical dimension of practical CPP-GMR sensors. To investigate the dependence of free layer thickness on the CPP-GMR reader’s stability performance, the output characteristics of the head, including the MR ratio, readback signal, dibit response, asymmetry parameter, and power spectral density (PSD) profile, were analyzed and discussed. The MR ratio basically represents the amplitude of the sensor’s output. The readback signal typically indicates the head response. It is obtained from the magnetization dynamic of the free layer passing through the Butterworth low-pass filter [36]. Based on the readback signal pattern, the dibit response is another important parameter indicating the nonlinear behavior and distortion occurring in the readback waveform. In this work, we performed the domain dibit extraction technique to obtain the linear dibit response as well as the nonlinearities via echoes around the main pulses [37]. In addition, an asymmetry parameter can be calculated from the difference between the positive and negative readback amplitudes, as written in Equation (1) [38].

(1) $$\%Asymmetry=\frac{\left(Vp-Vn\right)}{\left(Vp+Vn\right)}\times 100$$

The PSD profile demonstrates the fluctuation of the time-varying magnetization, as well as indicates the frequency spectrum of the readback signal. The local PSD is firstly calculated through the time-varying magnetization, ***M***_*x*,*y*,*z*_(***r***_i_,*t_j_*), where ***r****_i_* is the magnetization position at each varying time, *t_j_*, given in Equation (2) [39].

(2) $$S_{x,y,z}\left(r_{i},f\right)={\left|\underset{j}{\sum }M_{x,y,z}\left(r_{i},t_{j}\right)e^{i2\pi ft_{j}}\right|}^{2}$$

Then, the total PSD was computed by a summation of the local PSD at each particular frequency, *S*_*x*,*y*,*z*_(***r****_i_*, *f*), given as Equation (3). An integrated PSD can be further obtained by an integral of the overall PSD.

(3) $${\bar{S}}_{x,y,z}\left(f\right)=\underset{i}{\sum }S_{x,y,z}\left(r_{i},f\right)$$

## 4. Results and Discussion

In this section, the output characteristics of the CPP-GMR read head were characterized at different *t*_FL_ from 1 to 10 nm. The variation range of *t*_FL_ was based on the possible scale for practical devices while taking the covering trend of results into account. The focused parameters used for indicating the head stability performance, including the MR ratio, readback signal, dibit response, asymmetry parameter, and PSD profile, were analyzed and discussed.

**Figure 2:**
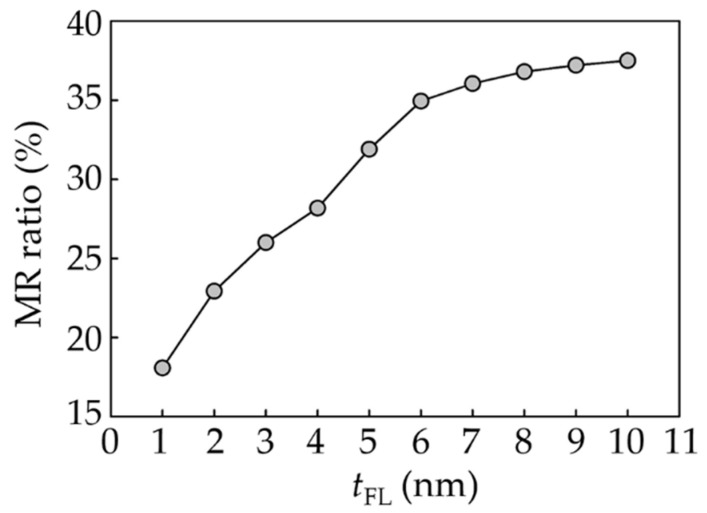
MR ratio of the CPP-GMR reader at various free layer thicknesses.

The MR ratio of the CPP-GMR reader versus *t*_FL_ is presented in Figure 2. It shows that the MR ratio increases at thicker *t*_FL_. Above a *t*_FL_ of 6 nm, a change in layer thickness has less impact on the MR ratio increment than below. An enhancement of bulk spin-dependent scattering contributed to an increase in MR ratio.

**Figure 3:**
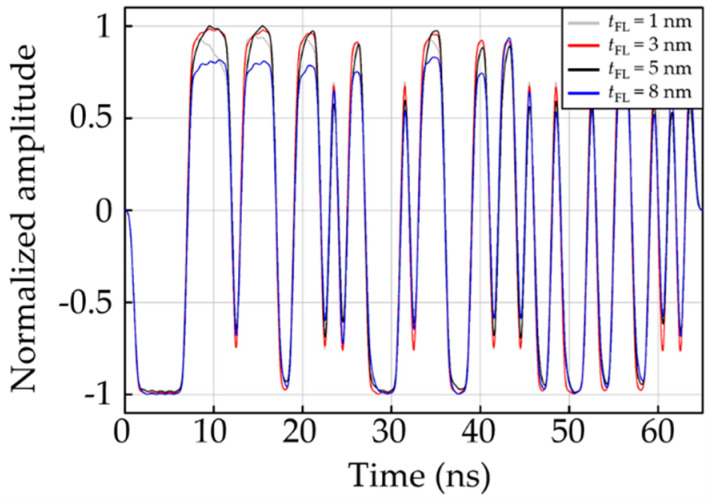
Examples of readback signal of the CPP-GMR reader.

To characterize the readback response of the CPP-GMR reader, we investigated the readback signal of the reader at *t*_FL_ of 1 to 10 nm. Examples of readback signal waveforms for *t*_FL_ of 1, 3, 5, and 8 nm are illustrated in Figure 3. The reader with a *t*_FL_ of 8 nm appears to have the highest distortion in the readback signal waveform. Meanwhile, the readback signals of the readers with *t*_FL_ of 3 and 5 nm are well patterned and symmetric. However, it is generally insufficient to analyze the readback response through only an investigation of the readback waveform. We therefore characterized more insights related to the readback signal behavior, which are the dibit response, asymmetry parameter, and the PSD profile. These parameters usually correspond to the stability performance of the read sensors. 

**Figure 4:**
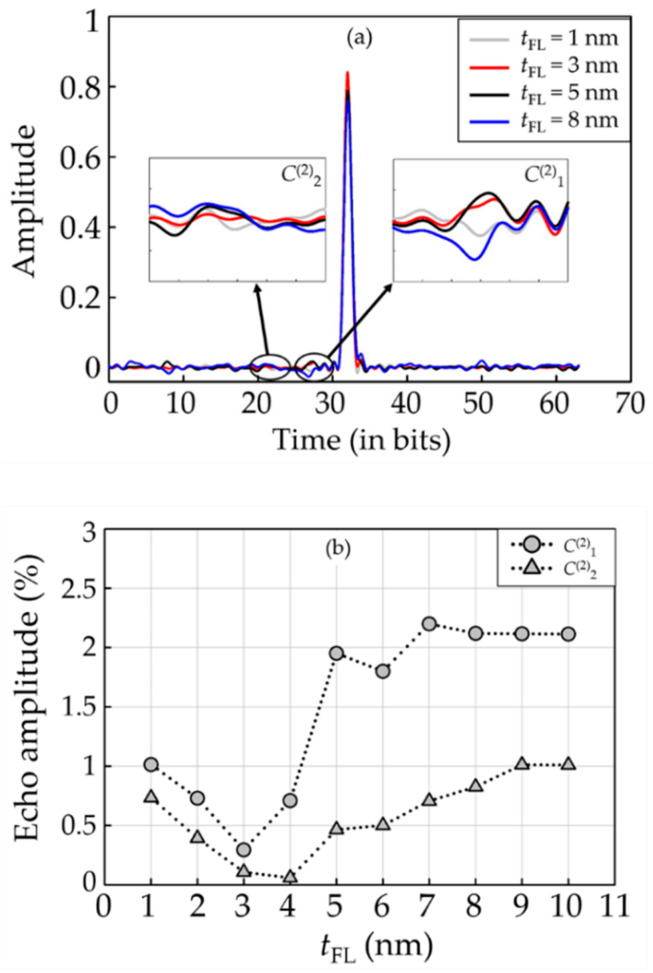
Dibit extraction of the CPP-GMR reader with different free layer thicknesses; (**a**) examples of dibit response; (**b**) amplitude of echoes.

The dibit response of the readback signal was obtained though the 63-bit PRBS with the polynomial *x*^6^ + *x*^5^ + 1. The main echoes related to this response are *C*^(2)^_1_ and *C*^(2)^_2_, which are located at bits 27 and 22, respectively. These echoes typically indicate the non-linear distortion of the readback waveform due to reader asymmetry [37]. It is noted that the impacts of higher orders of echoes were dominated by these main echoes and can be neglected in the evaluations. Figure 4a demonstrates the examples of dibit extraction of the reader with *t*_FL_ of 1, 3, 5, and 8 nm. Each echo is magnified in the insets. The readers having a *t*_FL_ of 5 and 8 nm seem to have higher echo amplitudes than others. Further details of echoes’ amplitudes were, in addition, analyzed at all possible *t*_FL_, as shown in Figure 4b. When the *t*_FL_ was increased starting from 1 nm, the amplitude of the echoes decreased until it reached its lowest scale. Then, the amplitude of echoes increased continuously when the *t*_FL_ increased beyond the point providing the lowest echo amplitude. A variation of *t*_FL_ appears to have a minor impact on *C*^(2)^_1_ and *C*^(2)^_2_ at a *t*_FL_ above 6 nm, indicating a lesser affectation on the readback signal distortion for this specific range.

**Figure 5:**
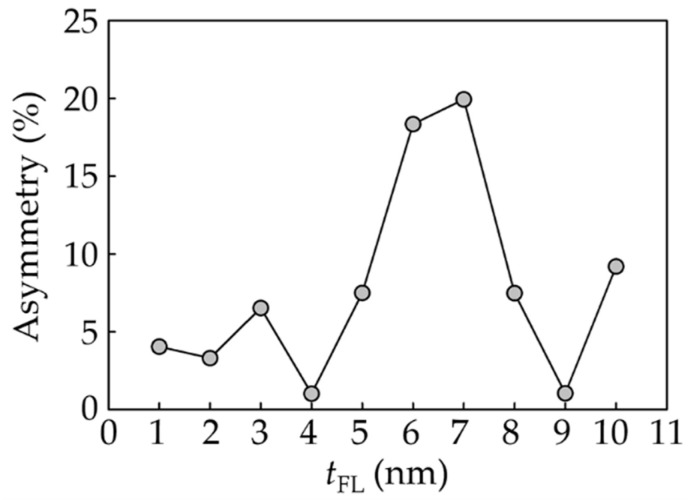
Asymmetry parameter obtained from the CPP-GMR reader’s response.

The asymmetry parameter of the readback signal obtained from the CPP-GMR reader was examined at different *t*_FL_, as shown in Figure 5. Most readers, except for those with a *t*_FL_ of 6 and 7 nm, were found to contain under 10% readback signal asymmetry. The readback waveforms produced by the readers with a *t*_FL_ of 4 and 9 nm are the most symmetric. From analysis of the readback signal, its dibit extraction, and its asymmetry parameter, it is obviously seen that the most suitable thickness of the free layer is 3 nm. The reader with this thickness value could provide the greatest pattern of readback signal.

**Figure 6:**
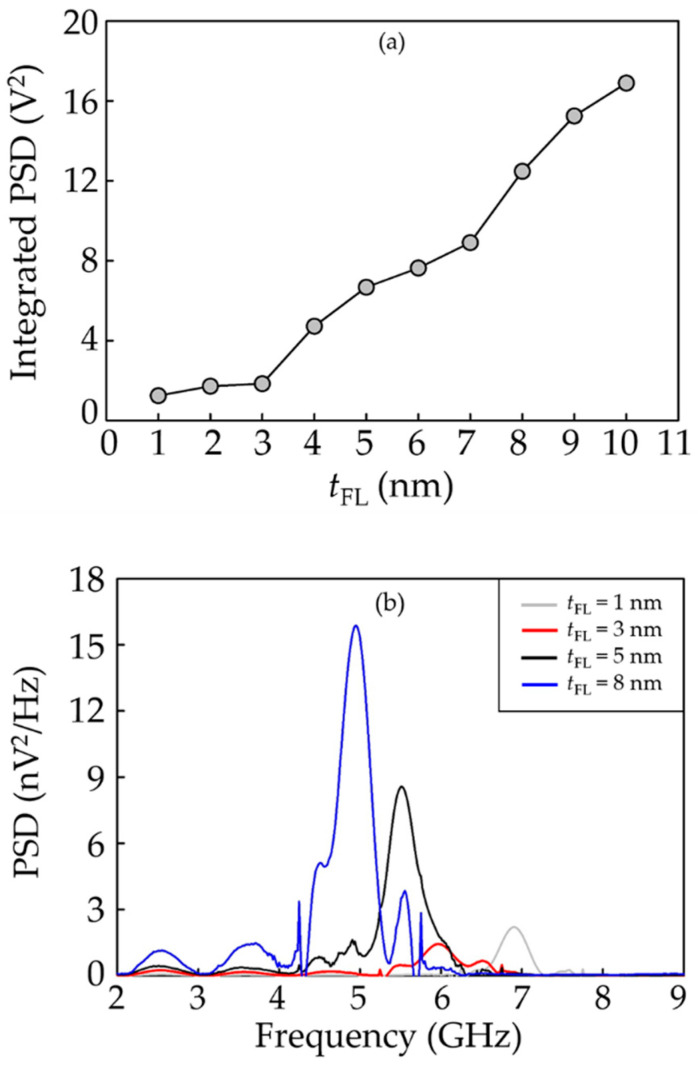
The PSD profile of the CPP-GMR reader at various free layer thicknesses; (**a**) integrated PSD; (**b**) frequency spectrum.

In addition, the PSD profile of the head response was characterized as another stability indicator for the reader. Figure 6a illustrates the integrated PSD at various *t*_FL_, while its frequency spectrum is indicated in Figure 6b. The PSD scale was found to be continuously lowered as the *t*_FL_ was reduced from 10 to 3 nm. Below *t*_FL_ = 3 nm, an adjustment of the *t*_FL_ causes a slight change in PSD amplitude. Then, it is worth reducing the thickness of the free layer to 3 nm. The frequency spectrum of PSD of the readers with *t*_FL_ of 1, 3, 5, and 8 nm is demonstrated in Figure 6b. Corresponding to Figure 6a, the amplitude of PSD becomes smaller at greater *t*_FL_. In particular, we found that the spectral peak is shifted to higher frequencies by decreasing the *t*_FL_. This frequency shifting behavior can be described by the magnetization precession which is computed by the LLGS equation [33,34]. The LLGS formula generally consists of the precession, damping, and spin torque terms. The spectral peak theoretically occurs depending mainly on the precession and damping terms of the time-varying magnetization. As the *t*_FL_ is reduced, the spin torque term in which its direction is opposing the magnetization precession becomes higher. This accordingly causes an enhancement of the force pulling the magnetization towards the opposite direction to its initial state. The resulting force therefore yields the higher oscillation of the magnetization precession. Rather than the frequency shifting, the higher PSD intensity at greater *t*_FL_ indicates a stronger impact on the reader stability, as this typically implies less stable magnetization precession and may further reduce the signal-to-noise ratio of the read sensors.

In summary, as the narrower physical reader gap of the read sensors is required to reach higher ADs, reducing the thickness of the free layer is therefore another approach to achieve this requirement. A very thin free layer, on the other hand, may result in an insufficient MR ratio. Then, in order to provide effective signal processing, an adequate MR ratio must be maintained. Our analysis shows that although a thinner free layer could provide a better readback response, the MR ratio is also reduced. Based on the trade-off between all characterized parameters, we believe that the appropriate *t*_FL_ of this particular CPP-GMR reader is 3 nm. At this point, a reasonable MR ratio of 26% is sufficient for practical devices. The highest stability performance of the reader with a *t*_FL_ of 3 nm was also confirmed through the analysis of the readback response that it is worthwhile to reduce the *t*_FL_ to 3 nm.

## 5. Conclusions

In this work, we investigated the dependence of the *t*_FL_ on the stability performance of the CMFG Heusler-based CPP-GMR sensor targeted for an areal density of 1 Tb/in^2^. Simulations were done based on micromagnetic modelling. It was found that the *t*_FL_ has a highly influential impact on the MR ratio at *t*_FL_ below 6 nm. A consideration of the readback signal of the head, including its dibit extraction and asymmetry parameter, indicates that the reader having a *t*_FL_ of 3 nm could produce a greatly patterned readback waveform. The PSD profile and its frequency spectrum are, in addition, analyzed and discussed to confirm the worthiness of setting the *t*_FL_ to 3 nm. Results also showed that a reasonably large MR ratio of 26% was greatly maintained at a *t*_FL_ of 3 nm. Therefore, the trade-off between all evaluated parameters suggests that this particular CPP-GMR reader with a *t*_FL_ of 3 nm indicates the best stability performance. Findings can be utilized to design the CPP-GMR reader for use in ultrahigh areal densities of magnetic data storage.
